# GradFreeBits: Gradient-Free Bit Allocation for Mixed-Precision Neural Networks

**DOI:** 10.3390/s22249772

**Published:** 2022-12-13

**Authors:** Benjamin Jacob Bodner, Gil Ben-Shalom, Eran Treister

**Affiliations:** Department of Computer Science, Ben-Gurion University, Beer Sheva 8410501, Israel

**Keywords:** neural network compression, quantization, gradient-free optimization, mixed-precision quantization

## Abstract

Quantized neural networks (QNNs) are among the main approaches for deploying deep neural networks on low-resource edge devices. Training QNNs using different levels of precision throughout the network (mixed-precision quantization) typically achieves superior trade-offs between performance and computational load. However, optimizing the different precision levels of QNNs can be complicated, as the values of the bit allocations are discrete and difficult to differentiate for. Moreover, adequately accounting for the dependencies between the bit allocation of different layers is not straightforward. To meet these challenges, in this work, we propose GradFreeBits: a novel joint optimization scheme for training mixed-precision QNNs, which alternates between gradient-based optimization for the weights and gradient-free optimization for the bit allocation. Our method achieves a better or on par performance with the current state-of-the-art low-precision classification networks on CIFAR10/100 and ImageNet, semantic segmentation networks on Cityscapes, and several graph neural networks benchmarks. Furthermore, our approach can be extended to a variety of other applications involving neural networks used in conjunction with parameters that are difficult to optimize for.

## 1. Introduction

Deep neural networks have been shown to be highly effective in solving many real-world problems. However, deep neural networks often require a large amount of computational resources for both training and inference purposes [1,2,3]. This limits the adoption and spread of this technology to scenarios with low computational resources.

To mitigate this computational burden, recent efforts have focused on developing specialized hardware to support the computational demands [4] as well as the model compression methods in order to reduce them [5]. These include various techniques such as pruning [6], knowledge distillation [7,8], a neural architecture search (NAS) [9], and as in this paper, quantization [10], which can naturally be combined with other approaches [11].

Quantization methods enable the computations performed by neural networks to be carried out with fixed-point operations rather than floating-point arithmetic [10,12,13]. This improves their computational efficiency and reduces their memory requirements. However, as with other compression methods, this typically comes at the cost of a reduced performance [5]. Recent efforts in the field have focused on improving the trade-offs between model compression and performance by proposing a plethora of quantization schemes tailored for different scenarios.

Quantization schemes can be divided into post-training and quantization-aware training schemes. Post-training schemes decouple the model training and quantization of its weights and/or activations and are most suitable when training data are not available when compressing the network [12,14,15,16]. Quantization-aware training schemes perform both optimization tasks together and do require training data, which tends to provide a better performance [17,18,19].

### 1.1. Paper Organization

The paper is organized as follows. In Section 1.2, Section 1.3, Section 1.4, Section 1.5, our problem definition, system model, and contributions are presented, followed by a comparison to the related works. The preliminaries to our method are then given in Section 2, the motivation for using the CMA-ES algorithm in our approach is given in Section 3.1, followed by the mathematical, algorithmic, and textual descriptions of our method, including the theoretical foundations, in Section 3.2, Section 3.3, Section 3.4, Section 3.5, Section 3.6. Finally, extensive experiments demonstrating the advantages of our approach are presented in Section 4, followed by ablation studies in Section 5 and conclusions and an outline of our future work in Section 6.

### 1.2. Problem Definition

In this paper, we focus on the problem of training quantized neural networks using mixed-precision quantization-aware training to improve the trade-offs between the performance and computational requirements of QNNs. The goal is to develop a training scheme that produces a fully trained QNN with optimal bit allocations per layer, according to the task and properties of the target edge devices. Furthermore, to ensure hardware compatibility, uniform quantization methods are preferred. Such methods divide the real-valued domain into equally sized bins, as in [18,19,20,21]. However, the proper allocation of bits between layers is combinatorial in nature and is hard to optimize. Furthermore, delicate interactions between weights and bit allocations must be considered to maximize the performance.

### 1.3. System Model

**Figure 1:**
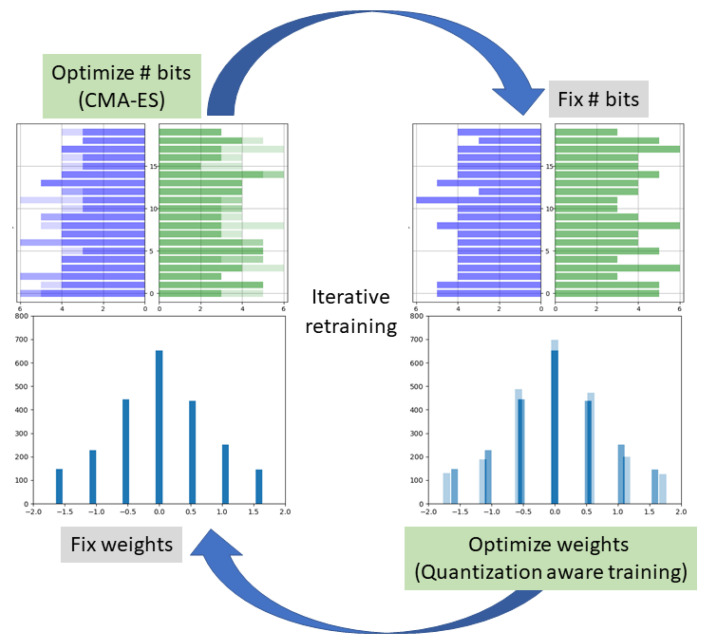
Our proposed training scheme: iterative optimization of the model weights and bit allocation. Given a fixed bit allocation (**right**), the weights are optimized using a gradient-based quantization-aware training procedure. Then, the weights are fixed (**left**) and the bit allocation is optimized for those weights using the CMA-ES [23] gradient-free optimization algorithm, and the training process is repeated in an iterative manner.

We propose a novel quantization-aware training procedure for uniform and mixed-precision QNNs, where a different number of bits is allocated per layer. In our training procedure, we utilize a gradient-based quantization-aware training procedure for the weights, and interchangeably, gradient-free optimization methods are used for computing the optimal bit allocation per layer for the weights and activations of the network. Such algorithms are known to perform well in difficult scenarios with complex dependencies between variables while maintaining an excellent sample efficiency during optimization [22]. In particular, we use the algorithm Covariance Matrix Adaptation Evolution Strategy (CMA-ES) [23], a highly versatile evolutionary search algorithm, which iteratively updates the parameters of a multivariate normal distribution to improve the fitness of the samples drawn from it. To summarize our approach, the network weights are updated by a gradient-based method while the bit allocation is updated using the gradient-free method CMA-ES—see Figure 1.

### 1.4. Our Contributions

The advantages of our approach are as follows:

- Our training scheme for mixed-precision QNNs optimizes the network as a whole. That is, it considers the dependencies between the layers of the neural network and the dependencies between the weights and their bit allocation.
- Our approach for optimizing the bit allocation is gradient free, and thus can handle multiple, possibly non-differentiable, hardware constraints. This enables tailoring QNNs to the resources of specific edge devices.
- We propose a bit-dependent parameterization for the quantization clipping parameters that allows for a better performance evaluation when sampling the network with a varying bit allocation.
- The systematic combination of gradient-based and gradient-free optimization algorithms can be utilized in other applications and scenarios, e.g., a search of the network’s other hyperparameters.

We demonstrate the performance of our method on popular tasks such as image classification and semantic segmentation, and also for graph node classification. For all test cases, our method achieves a better or on par performance with the current state-of-the-art low-precision methods and, in particular, yields a comparable accuracy for a lower model size when compared to a fixed-precision setting.

### 1.5. Related Works

#### 1.5.1. Fixed-Precision Methods

Most uniform per layer quantization methods rely on learned quantization parameters, such as the scaling parameters of the numbers before the rounding occurs. Quantization-aware training with fixed clipping parameters was initially proposed in [17], while the works [18,19,21] suggested ways to learn the clipping parameters. Several advances include weight normalization before quantization [20,24], a scale adjustment of the activations [19], soft quantization [25], and course gradient correction [26]. Recent efforts have focused on non-uniform quantization methods [27,28], which use lookup tables, making them difficult to deploy efficiently on the existing hardware. However, all the methods mentioned above use fixed-precision quantization (with the same bit allocation in all layers), which do not take into account the specific computational requirements and sensitivity to quantization noise that different layers may have.

#### 1.5.2. Mixed-Precision Methods

Recent efforts to tackle the mixed-precision quantization problem have included the use of reinforcement learning [29,30], a Hessian analysis [31,32], quantizer parametrization [33], and differentiable NAS approaches [34,35,36]. Among these methods, only the NAS approaches account for the dependencies between the bit allocations in the different layers by forming a super network that includes multiple branches for each precision at each layer. The NAS approaches, however, are often more expensive to train due to the multiple network branches which are used. Furthermore, they typically restrict their search spaces to a subset of bit allocations [35,36], which may harm the trade-offs between the performance and computational requirements.

#### 1.5.3. Joint Search Methods

A recent trend explores the joint search of mixed precision and architecture design to produce high-performance networks with low resource requirements. Such approaches however can be computationally expensive and often tailored to highly specific architectures [15,37,38,39]. However, joint mixed-precision and pruning methods are often not highly specific and can be applied to multiple architectures [40,41]. Though such methods reduce the number of operations performed, sparse operations and/or structured pruning are required to achieve measurable reductions in the computational cost.

## 2. Preliminaries

### 2.1. Quantization-Aware Training

In the uniformquantization scheme we consider, the real values of the weights are clipped between [−α,α] and the activations between [0,α]. As in [24], the range is mapped to the target integer range $\left[-2^{b-1}+1,2^{b-1}-1\right]$ for the weights and $\left[0,2^{b}-1\right]$, for the activations, where *b* is the number of bits. In this scheme, α, named “clipping parameters”, are trainable parameters that typically take on different values for different layers. Furthermore, to define the point-wise quantization operations used in our quantization-aware training scheme, we use two utility operations. The round(z) operation rounds all values in *z* to the nearest integer, and the clip(z,a,b) operation replaces all values z≤a with *a* and values z≥b with *b*. These are used in our point-wise quantization operations:

(1) $$\begin{array}{c} W_{b}=\alpha_{W}\frac{(round\left(\left(2^{b-1}-1\right)\cdot clip\left(\frac{W}{\alpha_{W}},-1,1\right)\right)}{2^{b-1}-1}, \end{array}$$

(2) $$\begin{array}{c} X_{b}=\alpha_{W}\frac{(round\left(\left(2^{b}-1\right)\cdot clip\left(\frac{X}{\alpha_{X}},0,1\right)\right)}{2^{b}-1}. \end{array}$$

Here, *b* is the number of bits that are used during quantization, $W,W_{b}$ are the real-valued and quantized weight tensors, $X,X_{b}$ are the real-valued and quantized input tensors, and $\alpha_{W},\alpha_{X}$ are their associated scale (or clipping) parameters, respectively. Equations (1) and (2) are used for training only, where the optimization of the weights and clipping parameters is obtained using the Straight-Through Estimator (STE) approach [24,42]. During the inference, the weights and activations are quantized, and all the operations are performed using integers in mixed precision while taking the clipping parameters into account. To improve stability, [20,24] also use weight normalization before quantization: $\hat{W}=\frac{W-\mu }{\sigma +\epsilon },$ where μ and σ are the mean and std of *W* respectively, and $\epsilon =10^{-6}$.

### 2.2. CMA-ES

Covariance Matrix Adaptation Evolution Strategy (CMA-ES) [23] is a population-based gradient-free optimization algorithm. It is known to be highly versatile and has been applied to a large variety of settings, such as reinforcement learning [43], placement of wave energy converters [44], hyperparameter optimization [45], and more. It is designed to work in *d*-dimensional continuous spaces and optimize discontinuous, ill-conditioned, and non-separable objective functions in a black-box optimization setting [46].

At a high level, at the *g*-th generation, the CMA-ES draws λ *d*-dimensional samples from a multivariate normal distribution $N(m^{\left(g\right)},C^{\left(g\right)})$:

(3) $$x_{k}^{\left(g+1\right)}∼m^{\left(g\right)}+\sigma^{\left(g\right)}N\left(0,C^{\left(g\right)}\right),fork=1,\ldots ,\lambda$$

where $m^{\left(g\right)}$, $C^{\left(g\right)}$, $\sigma^{\left(g\right)}$ are the mean, covariance matrix, and step-size used in the previous generation, respectively. Then, keeping only the top μ samples with the best objective values, $m^{\left(g+1\right)}$, $C^{\left(g+1\right)}$, and $\sigma^{\left(g+1\right)}$ of the next generation are calculated using a set of update rules [46]. This process is repeated until one of several stopping criteria are fulfilled. More details about CMA-ES are given in Appendix A.

## 3. The GradFreeBits Method

In this work, we base our *uniform* quantization-aware training scheme (for the weights and activations) on [24]. We apply a combination of gradient-based training rounds of the model weights while interchangeably applying gradient-free training rounds for optimizing the mixed-precision bit allocation, with CMA-ES. This process is referred to as iterative alternating retraining, illustrated in Figure 1.

### 3.1. Motivation: CMA-ES for Mixed Precision

We argue that CMA-ES is highly compatible with the problem of bit allocation in QNNs. We assume the objective function is the differentiable loss function used during training, with additional possibly non-differentiable constraints related to computational requirements (exact details appear below). As recent evidence suggests [30,31], the optimization landscape of the bit allocation problem is likely to be discontinuous, ill-conditioned, and amenable for optimization using gradient-free optimizers. Because the constraints may be non-differentiable, they can be sampled in a black-box setting, as is performed in gradient-free optimization (CMA-ES) and reinforcement learning [30]. Additionally, as shown in [31,32], the Hessian eigenvalues show large variations for different layers in QNNs, meaning that certain layers are typically more sensitive to changes in bit allocation than others. This is in part what motivated us to choose CMA-ES for this work, as it is capable of adapting to high variations in the Hessian eigenvalues and is therefore considered to be one of the best and widely used gradient-free methods.

### 3.2. Setting the Stage for CMA-ES

In order to optimize bit allocations of a QNN, two items must be defined: the search space and objective function.

#### 3.2.1. Search Space

We define the search space as a vector containing the bit allocation of the weights and activations in all the layers of the network, aside from the first and the last layers, as these are quantized using a fixed allocation of 8 bits. We found it beneficial to optimize the logarithm (base 2) of this vector rather than the vector itself. Thus, the vector to be optimized by CMA-ES is the log-precision vector:

(4) $$\begin{array}{cc} v_{W} & =\log_{2}\left(r_{W}\right),\\ v_{X} & =\log_{2}\left(r_{X}\right),\\ v & =[v_{W},v_{X}], \end{array}$$

where $r_{W},r_{X}$ are the bit allocations of the weights and activations, respectively, and [·,·] is the concatenation operation.

#### 3.2.2. Objective Function

Our objective function to minimize is a combination of the network’s performance measure, the differentiable loss function, subject to a number of possibly non-differentiable computational constraints:

(5) $$\begin{array}{cc} \min_{v} & =L(v;\theta ),\\ s.t. & \;h_{j}\left(v\right)\leq C_{j},\;forj=1,\ldots ,M, \end{array}$$

where L(v;θ) is the loss function over the training set, parameterized by network parameters θ, which are assumed to be fixed during the bit allocation optimization stage. Furthermore, $h_{j}\left(v\right)$ are the computational requirements for a given precision vector v (e.g., model size, inference time, etc.), $C_{j}$’s are the target requirements that we wish to achieve, and *M* is the number of constraints. To combine the constraints into the objective function, we use the penalty method:

(6) $$\min_{v}L\left(v;\theta \right)+\sum_{j=1}^{M}\rho_{j}\max{\left(0,h_{j}\left(v\right)-C_{j}\right)}^{2},$$

where $\rho_{j}$ are balancing constraint parameters. This is similar to the approach taken in [33], but here it is applied to gradient-free optimization. We define the computational constraints by matching the requirements of our mixed-precision network and to a fixed one. For example, we may require that the model size will be less than that of a fixed 4-bit allocation. We define a model size function MB(·) which takes in a log-precision vector for the weights and outputs the model size it produces when it was used to quantize the network. More formally, it is defined as follows:

(7) $$MB\left(v_{W}\right)=\sum_{i\in L}⎡2^{v_{W_{i}}}⎤|W_{i}|+\sum_{j\notin L}r_{j}\left|W_{j}\right|,$$

where ⎡*x*⎤ is the “ceil” operator, which rounds its argument up to the nearest integer, ⎡$2^{v_{W_{i}}}$⎤ is the precision used to quantize layer *i*, $|W_{i}|$ is the number of parameters in layer *i*, *L* is the set of layers to be quantized in mixed precision (all conv layers excluding the first). $r_{j}$ is the precision of layers not in *L*, specifically $r_{j}=8$ bits for first conv and last linear layers and $r_{j}=32$ bits for batch norm layers.

Using the notation in (4), we define the constraints on the model size for the weights entries $v_{W}$ and mean bit allocation for the activation entries $v_{X}$:

(8a) $$h_{1}\left(v\right)=MB\left(v_{W}\right),$$

(8b) $$C_{1}=\beta_{1}MB\left(v_{W}^{F}\right),$$

(9a) $$h_{2}\left(v\right)=\frac{1}{L}\sum_{i=1}^{L}v_{X_{i}},$$

(9b) $$C_{2}=\frac{\beta_{2}}{L}\sum_{i=1}^{L}v_{X_{i}}^{F},$$

Here, $v^{F}$ is the log-precision vector of the target fixed precision that we wish to achieve, and $v^{F}$ is the mixed log-precision vector. As in [24,33], MB(·) calculates the model size given weight entries $v_{W}$, *L* is the number of relevant layers, and $\beta_{1},\beta_{2}>0$ control the target compression rates of the weights and activations, respectively.

The constraints above are designed to limit the computational requirements while allowing the gradient-free optimization algorithm to explore non-trivial solutions which satisfy them. It is important to note that though these are mostly related to memory requirements, other constraints can easily be incorporated into our framework, such as power usage or inference time measurements, chip area, etc.

### 3.3. Gradient-Free Rounds

We define gradient-free steps as steps in which the CMA-ES algorithm optimizes the bit allocation (given the network’s weights) according to the objective function (6). In each gradient-free step, multiple generations of samples of the log-precision vector v are evaluated on (6). Because CMA-ES operates in continuous space, the bit allocations (positive integers) are extracted from v using: $r_{W}=⎡2^{v_{W}}⎤,r_{X}=⎡2^{v_{X}}⎤$. where ⎡*x*⎤ is the “ceil” operator. At each objective evaluation, the sample of v is used to quantize the weights and activations of the model. Then, the loss (6) is calculated over a set of minibatches, named a “super-batch”, yielding the value of the objective for each of the sampled bit allocations. Using this information, CMA-ES gradually minimizes the objective function, which enables non-trivial bit allocations to be found. In order to reduce subsampling noise during objective function evaluation, we define a moving super-batch as a set of minibatches, replaced in a queue-like manner. That is, in each iteration of the super-batch, we replace one of the minibatches within it. More details are given below in Section 3.5, and an ablation study on several replacement schemes is given in in our results section.

We define each gradient-free step to include a predefined number of objective evaluations *M*. It is important to note that gradient-free steps require significantly less computational resources than traditional epochs, even if the number of minibatches are matched. This is because they do not require backpropagation and the CMA-ES computations are negligible compared to forward passes of the model.

A gradient-free round is described in Algorithm 1, which applies several gradient-free steps utilizing the CMA-ES optimization algorithm. The terms $\theta ,v,v^{F}$ denote the network weights and the log-precision parameters of the mixed- and fixed-precision bit allocation, respectively. Furthermore, *d* is the number of log-precision parameters to optimize. First, the CMA-ES parameters $m^{\left(g\right)},\sigma^{\left(g\right)},C^{\left(g\right)}$ are initialized, then used to sample log-precision vectors in line (2), and are updated in line (11) according to the CMA-ES update rules. (See Appendix A for more details). In line (5), the network parameters θ and log-precision parameters v are inserted into the model and loss L(v;θ) is evaluated on the super-batch.
**Algorithm 1** Gradient-Free Rounds.
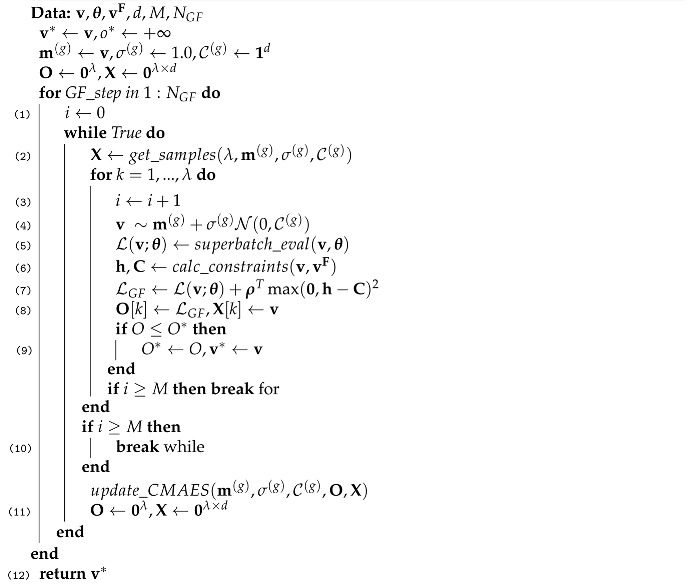


### 3.4. Iterative Alternating Retraining

To start the optimization process, the model is pretrained with the quantization-aware training scheme in Section 2.1, using a fixed bit allocation. After this stage, the model is passed to the gradient-free optimizer CMA-ES to optimize its bit allocation for a round of $N_{GF}$ steps, as described in Algorithm 1. This adapts the bit allocation to the model weights, which are fixed at this stage in their floating-point values, and enables CMA-ES to maximize the performance of quantized networks, subject to the computational constraints (Equation (6)). Once the gradient-free round is completed, the bit allocation with the lowest objective value is passed to the gradient-based optimizer for a gradient-based round of $N_{GB}$ epochs. This adapts the model weights to the bit allocation, which is kept fixed, using the quantization-aware training scheme described in Section 2.1. The cycle is repeated several times until the performance and computational requirements are satisfactory. The process is illustrated in Figure 1. The output of this process is a fully trained mixed-quantization model, which can be readily deployed on the target edge device.

### 3.5. Variance Reduction in CMA-ES Sampling

Variance reduction has been shown to improve the convergence rate of optimization algorithms [47]. The main source of variance in our objective function (Equation (6)) is in the first term, related to the performance of the model for different bit allocations. There are two main causes of variance in this term: subsampling noise, caused by using small minibatches of randomly selected samples, and sensitivity to quantization errors, which networks are typically not robust to. In this section, we propose a mitigation to the first cause of variance, while in the next section, we propose a mitigation for the second.

#### Moving Super-Batches

**Figure 2:**
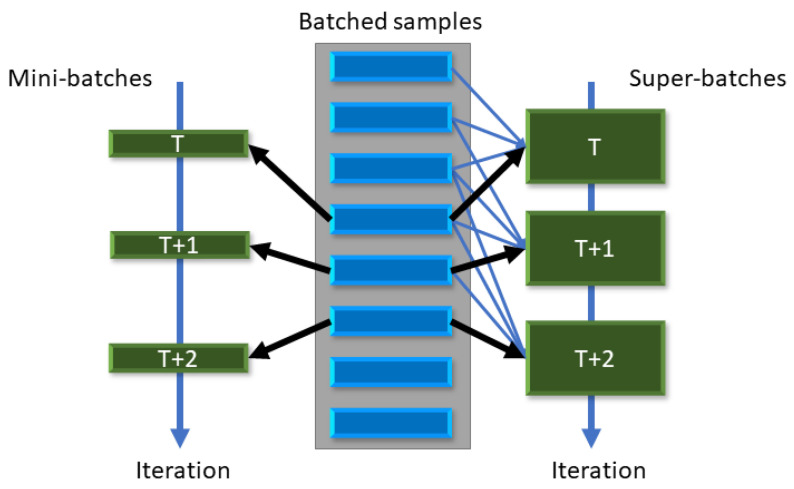
Batch replacement scheme used in moving super-batches, as compared to standard minibatch replacement. At each iteration, a single minibatch is replaced, in a queue-like manner, creating a high overlap of samples between consecutive super-batches.

To mitigate subsampling noise in our objective function (Equation (6)), we define a moving super-batch as a set of minibatches which are replaced in a queue-like manner. That is, in each iteration of the super-batch, we replace part of the minibatches within it. Figure 2 illustrates this approach. During each objective evaluation of CMA-ES, the entire super-batch is run through the model in order to calculate the first term of (6). The queue-like replacement scheme enables CMA-ES to encounter new data samples in each objective evaluation but with a larger overlap of data samples as compared to SGD, where the minibatches are re-sampled at each iteration. Several strategies for the frequency of replacement can be considered, such as replacing one or more minibatches after each objective evaluation, or doing so every fixed number of evaluations. These different settings are explored in the ablation study in Section 5.2.

### 3.6. Adapting the Clipping Parameters to Varying Bit Allocations

Any alternating minimization scheme that operates similarly as described above has the following shortcoming. Sampling the loss of the network with a set of new bit allocations that are incompatible with the training of the other parameters may lead to a misguided measure of performance. The most significant effect is due to the clipping parameters $\alpha_{X}$ and $\alpha_{W}$ in (1) and (2). To this end, we parameterize the clipping parameters and train the network’s weights to be compatible with multiple bit allocations through stochastic choice of bit allocations during training. Because the weights throughout the optimization process are in floating point, we can apply their quantization with different bit allocations at each time but need the clipping parameters to be automatically adapted to the bit allocation. To support this desirable property, we follow the analysis in [12] regarding the influence of the bit allocation on the optimal clipping parameters.

According to [12], for a Laplace (0,β) distribution and $2^{b}$ quantization intervals, the quantization noise is given by

(10) $$E\left({\left(W-W_{b}\right)}^{2}\right)\approx 2\beta^{2}\cdot \exp\left(-\alpha /\beta \right)+\frac{\alpha^{2}}{3\cdot 2^{2b}}$$

and therefore,

(11) $$\frac{\partial E({\left(W-W_{b}\right)}^{2})}{\partial \alpha }\approx -2\beta \cdot \exp\left(-\alpha /\beta \right)+\frac{2\alpha }{3\cdot 2^{2b}}.$$

Setting (11) to zero led [12] to an optimal clipping parameter in post-training quantization, while here we use it to obtain a relation between the optimal clipping parameter with the number of bins used for the quantization:

(12) $$\frac{\alpha }{\beta \cdot 3\cdot 2^{2b}}=\exp\left(-\alpha /\beta \right).$$

Setting a log on the two side yields:

(13) $$\begin{array}{cc} \frac{\alpha }{\beta }+\log\left(\frac{\alpha }{\beta }\right) & =\log(3\cdot 2^{2b}),\\ \alpha +\beta \log(\frac{\alpha }{\beta }) & =c\cdot b, \end{array}$$

where *b* is the number of bits, and *c* is a constant.

There is no closed-form solution to Equation (13), and generally, it is involved with a few possibly inaccurate assumptions. First, the derivation of (10) is only approximated. Second, the assumption of the weights or activations being drawn from a Laplace distribution is reasonable but not entirely accurate. Third, the scale β is unknown, and lastly, the MSE is a reasonable error measure but not necessarily the best measure for optimizing a multi-layer neural network. Hence, we do not solve (13) directly but use it as guidance for parameterizing the clipping parameters α(b).

**Figure 3:**
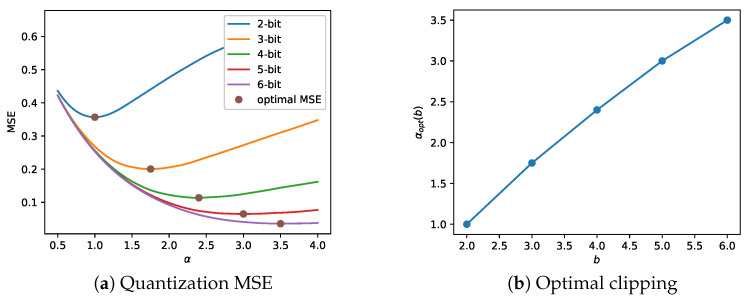
The quantization MSE and the associated optimal clipping parameter $\alpha_{opt}\left(b\right)$ for samples drawn from a Laplace (0, 0.5) distribution. The plot in (**a**) shows the MSE per clipping parameter α and the optimal α’s for each bit allocation *b*. The plot in (**b**) shows a linear relation between $\alpha_{opt}$ and the number of bits *b*.

To deal with the choice of the clipping parameter α, we adopt the gradient-based optimization described in Section 2.1, but we parameterize it to account for different bit allocations. Specifically, we use a simple linear approximation

(14) $$\alpha \left(b\right)=\alpha^{\left(0\right)}+\alpha^{\left(1\right)}b,$$

instead of each of the parameters in (1) and (2). This is a reasonable choice in the premise of the assumptions above (Laplace distribution, MSE as error measure)—see Figure 3. Different assumptions on the distribution (e.g., normal distribution), error measure (e.g., mean absolute error), and similar uniform quantization schemes yield similar results. We note that the relation is not exactly linear and may benefit from a richer parameterization than in (14).

The parameterization in Equation (14) is used for the quantization in (1) and (2). To train $\alpha^{\left(0\right)},\alpha^{\left(1\right)}$ for each layer, we perturb the bit allocation during the pretraining stage, randomly changing per layer bit allocations by +1, −1 or 0 bits (no-change), around the predefined fixed bit allocation in pretraining stages. Finally, we also conduct an ablation study to verify the expected performance gains throughout the training. (See Section 5).

## 4. Experiments and Results

To quantitatively compare our mixed-precision scheme (GradFreeBits) to other related works, we apply it to several neural network architectures for image classification, semantic segmentation, and semi-supervised graph node classification tasks. The properties of the datasets and training configurations of the image datasets are detailed in Table 1. Throughout all the experiments, we use the bit-dependent clipping parameters described in Equation (14). For the mixed case, the averaged number of bits across the layers is considered. We compare our approach to the related works that use uniform quantization, with either fixed (F) or mixed (M) bit allocation schemes, which quantize *both* the weights and activations. For the ImageNet and segmentation encoder models, we used pretrained weights from TorchVision [48]. Our code is written in the PyTorch framework [49], and the experiments were conducted on an NVIDIA RTX 2080ti GPU.

### 4.1. CIFAR 10/100

The CIFAR10 and CIFAR100 image classification benchmarks [50] have 10 and 100 classes, respectively. The full dataset properties and training configuration can be found in Table 1. Additionally, we used random horizontal flips and crops and mixup [53] data augmentations.

The results for the CIFAR10 dataset are presented in Table 2. Our method outperforms the previous state-of-the-art EBS(M) for both the ResNet20 and ResNet56 models at both mixed-precision settings, e.g., +0.5% for 4-bit ResNet20 and +0.5% for 4-bit ResNet56. Table 2 also includes the results for the CIFAR100 dataset, in which our method also outperforms all the other related works by +1.6% for 4-bit ResNet20 and +0.7% for 3-bit ResNet20. We believe that our method outperforms the related methods because it considers a larger search space of bit allocations compared to the other methods, which limit the search space [34,36] or use a fixed bit allocation [17,18,20,21,26]. For example, we consider 1–8 bits for the weights and activations in each layer, a total of 64 combinations, while [36] uses a set of 8 such manually selected combinations. Even though the search space is larger, our method is able to efficiently optimize it due to the excellent sample efficiency of the CMA-ES [23].

### 4.2. ImageNet

The ImageNet [51] image classification benchmark has 1 K classes and 1.2 M train and 150 K test RGB images. The full dataset properties and training configuration can be found in Table 1. The data augmentations are identical to those used in the CIFAR10/100 experiments (above).

The results for the ImageNet dataset are presented in Table 3. Our ResNet18 model achieves the highest Top1 accuracy and the lowest model size compared to all the other methods in 2W/2A–3W/3A. For example, our 3W/3A ResNet18 model achieves a smaller model size of −0.2 MB and a higher Top1 accuracy of +0.5 compared to next best *uniform* APoT [24]. For the ResNet50 model, our method consistently achieves the smallest model size, with a comparable Top1 accuracy to the other methods. Our method achieves the smallest model sizes −1 MB, −0.3 MB at the cost of a slightly reduced performance −0.2, and −0.6, compared to [19,36], for the 3W/3A and 4W/4A models, respectively. However, for the 2W/4A model, our method achieves a slightly worse Top1 accuracy of −0.3 compared to [32], though at a significantly smaller model size of −4.9 MB (a 37.4% reduction). For the 2 W/2 A model, our method achieves the highest accuracy in this category, +1.0 compared to [19], despite having the same model size. We believe that the improvements in the trade-offs are primarily due to our use of larger search spaces, 64-bit combinations for each layer, compared to the 49, 8, and 6 combinations in [30,35,36], respectively. Our method is able to properly optimize the bit allocations in this large search space as it uses the CMA-ES which has an excellent sample efficiency [23]. Furthermore, our method does not make simplifying assumptions regarding the interactions between the layers, as used in [31,32], which again increases the search space and enables our method to find superior bit allocations.

We also provide a cost comparison with the other fixed- and mixed-precision quantization methods, in terms of the number of epochs, in Table 3. Using the experimental details for ImageNet from Table 1, we find that the GFB uses only 57 effective epochs: 30 quantization-aware training epochs and 27 effective epochs for the bit optimization. Because the gradient-free steps have similar costs to the gradient-based epochs (see Appendix B), our training cost is calculated using five gradient-based epochs plus four gradient-free steps applied iteratively for three rounds: effective_epochs=3×(4+5)=27. More details can be found in Appendix B.

Our method requires the lowest computational cost compared to all the other methods, which typically require 100–200 epochs. We believe that our improved training costs may be due to the excellent sampling efficiency of the CMA-ES, which is able to find the optimal bit allocations in a relatively conservative budget of gradient-free steps.

### 4.3. Image Semantic Segmentation

In this section, we demonstrate the advantage of mixed precision for semantic segmentation, which is another common task for low-resource devices, such as automobiles, robots, and drones. Semantic segmentation is an image-to-image task where every pixel needs to be classified. Hence, this task is much more sensitive for quantization compared to the classification results above, where the network produces a single class [54]. We present our results on the popular Cityscapes dataset [52]. The dataset properties and training procedure are described in Table 1. We adopt the popular segmentation architectures DeepLabV3 [55] and DeepLabV3+ [56] with ResNet50 [57] and MobileNetV2 [58] encoders, and with the standard ASPP [55] module for the decoder. For a fair comparison to [16], the images are resized to 256, with random horizontal flips, during training.

In Table 4, we compare our method to the *fine-tuned* models in [16], where the data were available for training. Even though the method of [16] uses knowledge distillation, our method achieves a strictly superior model size and mIoU in all bit allocations. This is best seen in our 8-bit DeepLabV3(ResNet50), which achieves a +2.5 mIoU despite having a significantly smaller model size of −7.3 MB.

**Figure 4:**
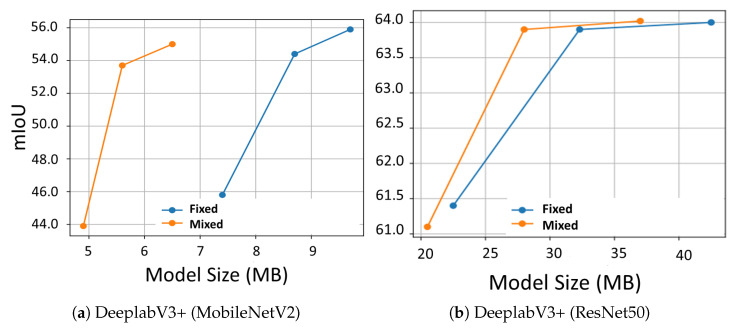
mIoU vs. model size on Cityscapes for different quantized models using fixed precision in 4, 6, and 8 bits and GFB mixed-precision counterparts. (**a**) Quantized DeeplabV3+ with MobileNetV2 backbone, (**b**) quantized DeeplabV3+ with ResNet50 backbone.

**Figure 5:**
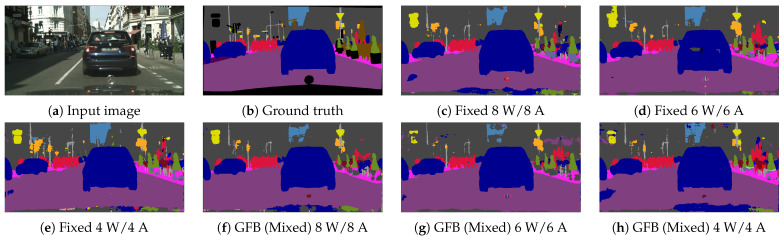
Sample segmentation results from Cityscapes using quantized DeepLabV3+ (ResNet50). (**a**) Input image. (**b**) Ground truth. (**c**–**e**) present outputs of 8-, 6-, and 4-bit fixed quantization models respectively. (**f**–**h**) present outputs of 8-, 6-, and 4-bit mixed quantization models, respectively.

We also provide comparisons between our fixed- and mixed-precision models in Figure 4, as well as sample images in Figure 5. The results clearly demonstrate the added value of mixed over fixed quantization models, as the former typically provides better trade-offs between the model size and performance. For example, in Figure 4a, our mixed quantization DeepLabV3+ (MobileNet) models typically achieve a 30% improvement in model size, though with a small degradation of −1.5 in the mIoU, a better trade-off than is obtained by reducing the precision of fixed quantization models. For the DeepLabV3+ (MobileNet) in Figure 4b, the improvement in trade-offs is smaller, though still apparent. Furthermore, the sample images in Figure 5 demonstrate that though the quality of the segmentation maps clearly degrades as the network is quantized to a lower precision, it is evident that the mixed-precision model (Figure 5f,g) provides more accurate segmentation maps than the fixed-precision model (Figure 5c–e). For example, the shape of the “yield” sign in the top right part of Figure 5b is better preserved by the mixed-precision model (Figure 5f,g).

In this section, we demonstrate an application to semi-supervised node classification, as the compression of graph neural networks is important for several real-world applications, such as in autonomous vehicles. We compare the GFB to [59], using the GCNII [60] with 32 layers, on three common semi-supervised node classification datasets: Cora, Pubmed, and Citeseer [61]. The details of these datasets are provided in Table 5. The training procedure uses the following hyperparameters. In all the experiments, we used 8 bits for the first and last layers, an SGD with lr=0.01, momentum=0.9 cosine scheduler, $\beta_{1},\beta_{2}=0.98$, $\rho_{1},\rho_{2}=10.0,M=1024,N_{GF}=5,N_{GB}=4,N_{Rounds}=3$, 30 pretrain epochs, and v s. space limited to [0.0−3.6]. We compare to the GCNII in [59] which uses only quantization and no wavelet compression and uses 8-bit weights while reducing the activations precision.

The results for the semi-supervised node classification are reported in Table 6. The GFB achieves a significantly higher accuracy for comparable compression rates. For example, in the 8W/2A bit Pubmed, the GFB achieves a significantly higher accuracy (+37.1), compared to [59], even when considering the differences in the 8W/8A baselines. We believe this may be due to a higher observed bit allocation in the initial graph convolution layers, which tend to have a greater effect on the performance of the GCNII, compared to the deeper layers. This is due to the design of the GCNII, which reduces the effects of the deeper layers on the outputs to avoid the oversmoothing phenomena observed in the original GCN [60].

## 5. Ablation Study

### 5.1. Bit-Dependent Clipping Parameters

**Figure 6:**
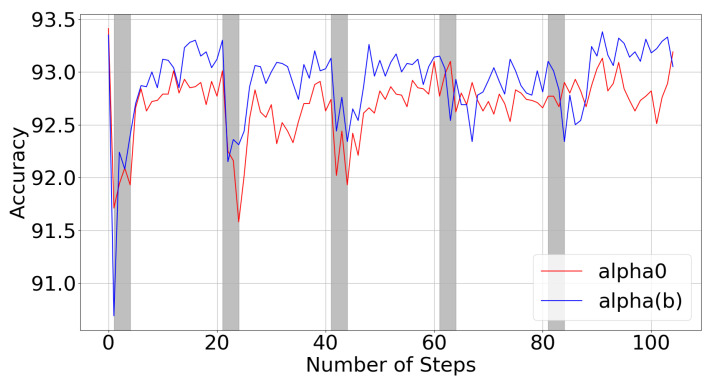
Accuracy during iterative alternating retraining stage, using different clipping parameter bit dependencies, for 4-bit mixed-precision ResNet20 on CIFAR10. Grey regions correspond to gradient-free steps, while white regions correspond to gradient-based epochs.

We examine the effects of the fixed vs. bit-dependent clipping parameters (Equation (14)) which were pretrained using perturbed bit allocations around the predefined fixed bit allocation. The results are displayed in Figure 6. The fixed clipping parameters (red) tend to perform slightly worse than the bit-dependent clipping parameters (blue). As expected, they lead to larger performance drops during the gradient-free rounds (grey), where the different bit allocations are evaluated and optimized. Moreover, the latter seems to have a lower variance. We believe the variance reduction is caused by the noise forcing the network to learn the proper relationships of α(b), or the robust values of α, rather than an arbitrary combination of $\alpha^{\left(0\right)}$ and $\alpha^{\left(1\right)}$ which provide a low training loss for only the given fixed bit allocation.

### 5.2. Iterative Alternating Retraining

Here, we examine the effects of pretraining, iterative alternating retraining, and the number of minibatches they contain. All the experiments are conducted with 4-bit mixed-precision ResNet20 models on CIFAR100 with the same hyperparameters used in Section 4.1.

The results of the ablations study are presented in Table 7. The −3.6% accuracy degradation demonstrates that pretraining plays a crucial role in reducing the performance degradation due to the changes in the bit allocation. It also seems that iterative alternating retraining, as opposed to separating the bit optimization and weight optimization stages, leads to a small +0.2% increase in performance, demonstrating the added value of this approach. Regarding the super-batch settings, it seems that the optimal setting is to use 32 minibatches and replace a single batch after each objective evaluation (SB), also leading to a performance increase of +0.2%.

## 6. Conclusions

We proposed GradFreeBits, a novel framework for mixed-precision neural networks, which enables customization to meet multiple hardware constraints. The framework is based on the combination of a gradient-based quantization-aware training scheme for the weights and gradient-free optimization of the bit allocation, based on the CMA-ES. The combination of the two approaches in an iterative alternating retraining scheme is quite general, making it likely easy to extend to other applications. Additionally, we propose a novel parameterization of the clipping parameters to facilitate their adaptation to different bit allocations.

Through extensive experimentation, we find that our method achieves superior or comparable trade-offs between the accuracy and model size, though at lower training costs, compared to several mixed and fixed quantization methods on a wide variety of tasks, including image classification, image semantic segmentation, and (graph) semi-supervised node classification benchmarks. Furthermore, we find that our proposed bit-dependent clipping parameters provide measurable gains in the performance of mixed-precision models, with negligible added parameters.

Future work includes utilizing additional constraints, such as measurements from hardware simulators. Additionally, we believe that extending our iterative retraining approach to new scenarios, such as optimizing per layer pruning rates, may provide similar benefits in trade-offs between accuracy and computational cost.

## Figures and Tables

**Table 1:** Image dataset properties and training configurations. v s. space denotes log-precision vector search space used by CMA-ES and Bit s. space are corresponding bit allocations. * Original images are resized to the size provided using bilinear interpolation.

Property	C10/100	ImageNet	Cityscapes
	[50]	[51]	[52]
# Train	50 K	1.2 M	2975
# Test	10 K	150 K	1525
# classes	10/100	1000	19
Img size	32	224 *	256 *
Batch size	128	100	4
# Batch/S-Batch	32	16	16
Optimizer	SGD	SGD	SGD
lr-enc	0.1	0.001	$10^{-4}$
lr-dec	-	-	0.1
Momentum	0.9	0.9	0.9
$\beta_{1}$	0.9	0.9	0.98
$\beta_{2}$	0.98	0.9	0.98
$\rho_{1}$	20.0	10.0	0.1
$\rho_{2}$	0.5	10.0	0.5
Pret. epochs	300	30	80
*M*	1024	1024	512
$N_{GF}$	4	4	4
$N_{GB}$	16	5	16
$N_{Rounds}$	5	3	3
v s. space	[0.0–3.0]	[0.0–3.0]	[0.0–3.6]
Bit s. space	1-8b	1-8b	1-12b

**Table 2:** Top1 accuracy on CIFAR10/100. (F) and (M) denote fixed and mixed precision.

CIFAR-10 with ResNet20, FP Accuracy 93.3%			
Method	2W/4A	3W/3A	4W/4A
PACT(F) [18]	-	91.1	91.7
LQN(F) [21]	-	91.6 -	-
BCGD(F) [26]	91.2	-	92.0
DQ(M) [33]	91.4	-	-
HAWQ(M) [31]	92.2	-	-
EBS(M) [34]	-	92.7	92.9
BPNAS(M) [36]	-	92.0	92.3
GFB(M) (ours)	93.0	93.2	93.4
**CIFAR10 with ResNet56, FP Accuracy 95.1%**			
EBS(M) [34]	-	94.1	94.3
GFB(M) (ours)	-	94.7	94.8
**CIFAR100 with ResNet20, FP Accuracy 70.35%**			
DRFN(F) [17]	-	68.4	68.9
LQN(F) [21]	-	68.4	69.0
WNQ(F) [20]	-	68.8	69.0
GFB(M) (ours)	-	69.6	70.6

**Table 3:** Top1 accuracy on ImageNet. (M) denotes mixed precision, (·) denotes model size, measured in MB. Subscripts denote reported difference in accuracy, compared to the FP accuracy reported in the original papers. * identifies methods that *do not quantize* the first and last layers. The cost of each method is presented as the total number of epochs required for pretraining, search, and fine-tuning. ∼X presents estimated cost obtained from text descriptions in the original papers.

Method	2 W/2 A	2 W/4 A	3 W/3 A	4 W/4 A	32 W/32 A	Cost (# Epochs)
ResNet18						
PACT [18]	$64.4_{-6.0}$ (3.2)	-	$68.1_{-2.3}$ (4.7)	$69.2_{-1.2}$ (6.1)	70.4 (46.8)	110
DSQ [25]	$65.2_{-4.7}$ (3.2)	-	$68.7_{-1.2}$ (4.7)	$69.6_{-0.3}$ (6.1)	69.9 (46.8)	-
APoT [24]	-	-	$69.4_{-1.3}$ (4.7)	-	70.7 (46.8)	120
SAT [19]	$65.5_{-4.9}$ (3.2)	-	$69.3_{-0.9}$ (4.7)	$70.3_{+0.1}$ (6.1)	70.2 (46.8)	150
DQ(M) [33]	-	-	-	$70.1_{-0.2}$ (5.4)	70.3 (46.8)	160
SPOS * (M) [35]	$66.4_{-4.0}$ (-)	-	$69.4_{-1.0}$ (-)	$70.6_{+0.2}$ (-)	70.4 (46.8)	240
GFB(M) (ours)	$66.5_{-3.9}$(3.2)	-	$69.9_{-0.5}$ (4.5)	$70.3_{-0.1}$ (5.4)	70.4 (46.8)	57
ResNet50						
PACT [18]	$72.2_{-4.7}$ (8.1)	-	$75.3_{-1.6}$ (11.0)	$76.5_{-0.4}$ (13.9)	76.9 (102.2)	110
SAT [19]	$73.3_{-2.6}$ (8.1)	-	$75.9_{-0.0}$ (11.0)	$76.3_{+0.4}$ (13.9)	76.7 (102.2)	150
BPNAS(M) * [36]	-	-	$75.7_{-1.9}$ (11.3)	$76.7_{-0.7}$ (13.4)	77.4 (102.2)	150
HAQ(M) * [30]	-	$75.5_{-0.6}$ (12.2)	-	-	76.2 (102.2)	-
HAWQ(M) * [31]	-	$75.5_{-1.9}$ (13.2)	-	-	77.4 (102.2)	∼400
HAWQV2(M) * [32]	-	$75.8_{-1.6}$ (13.1)	-	-	77.4 (102.2)	∼400
GFB(M) (ours)	$74.3_{-2.1}$ (8.1)	$75.5_{-0.9}$ (8.2)	$75.7_{-0.7}$ (10.7)	$76.1_{-0.3}$ (12.8)	76.4 (102.2)	57

**Table 4:** Mean intersection over union (mIoU) of quantized DeepLabV3(ResNet50) on Cityscapes. FP mIoU: 64.7, FP model size: 158.5 MB. For DeepLabV3+ (ResNet50), the FP mIoU and model size are 64.7 and 159.1 MB, respectively. (F) and (M) denote fixed and mixed precision, (·) denotes model size, measured in MB. HR denotes DeepLabV3 (ResNet50) models trained with images resized to 512 rather than 256, hence the accuracy is higher.

Bits	ZAQ-FT (F)	GFB (M)	GFB-HR (M)
W/A	[16]	(Ours)	(Ours)
4/4	56.0 (22.1)	62.5 (21.2)	71.6 (22.0)
6/6	59.6 (31.8)	62.9 (30.2)	71.4 (27.6)
8/8	61.2 (41.6)	63.7 (34.3)	72.7 (38.5)

**Table 5:** Dataset statistics of semi-supervised node classification benchmarks.

Data	Classes	Label Rate	Nodes	Edges	Features
Cora	7	0.052	2708	5429	1433
Citeseer	6	0.036	3327	4732	3703
Pubmed	3	0.003	19,717	44,338	500

**Table 6:** Classification accuracy of quantized GCNII in semi-supervised node classification. F/M denotes fixed-precision and mixed-precision results, respectively.

Benchmark	Bits	GFB (M)	Quantized GCNII [59] (F)
Cora	8W/8A	83.7	80.9
FP Acc 85.4	8W/4A	83.1	31.9
	8W/2A	81.2	21.1
Citeseer	8W/8A	72.0	69.8
Acc 73.2	8W/4A	71.8	24.7
	8W/2A	70.3	18.3
Pubmed	8W/8A	79.6	80.0
Acc 80.3	8W/4A	79.6	41.3
	8W/2A	77.8	40.7

**Table 7:** Top1 accuracy of 4-bit mixed-precision ResNet20 on CIFAR100, for various system settings. We use the following shorthand: “SS.” for super-batch setting, “|B|” for number of minibatches in the super-batch, “IAR.” for iterative alternating retraining, “PRET.” for pretraining.

Variable	|B|	IAR.	PRET.	Top1 Acc.
Baseline	32	*√*	*√*	70.61
Components	32	*√*	×	66.99
	32	×	*√*	70.33
Super-batch	4	*√*	*√*	69.68
Size	8	*√*	*√*	70.25
	16	*√*	*√*	70.18
	64	*√*	*√*	70.26

## Data Availability

In this paper, we use several publicly available datasets. For the image classification experiments, we use the CIFAR10/100 dataset [50], available at https://www.cs.toronto.edu/~kriz/cifar.html (accessed on 23 January 2019), and the ImageNet dataset [51], available at https://www.image-net.org/ (accessed on 15 January 2019). For the semantic segmentation experiments, we use the Cityscapes dataset [52], available at https://www.cityscapes-dataset.com/ (accessed on 12 May 2019). Finally, for the semi-supervised node classification experiments, we use the Cora, CiteSeer, and Pubmed datasets [61], all available through torch geometric [62], at https://pytorch-geometric.readthedocs.io/en/latest/modules/datasets.html (accessed on 7 October 2021), under the “Planetoid” dataset.

## Appendix A. CMA-ES

In this section, we provide a very brief overview of the main derivations that are used in the CMA-ES, without including any theoretical background for the structure of these update rules, or the choices of hyperparameters. For more details regarding these topics, we refer the curious reader to [63]—we follow the same notation as this paper here.

Covariance Matrix Adaptation Evolution Strategy (CMA-ES) [23] is a population-based gradient-free optimization algorithm. At a high level, the optimization process of the CMA-ES is as follows. At the *g*-th generation, a set of λ *d*-dimensional samples $x_{k}\in R^{d}$ are drawn from a multivariate normal distribution $N(m^{\left(g\right)},C^{\left(g\right)})$:

(A1) $$x_{k}^{\left(g+1\right)}∼m^{\left(g\right)}+\sigma^{\left(g\right)}(N0,C^{\left(g\right)}),fork=1,\cdots ,\lambda$$

where $m^{\left(g\right)}$, $C^{\left(g\right)}$ are the mean and covariance matrix of the population at the previous generation, respectively. λ is the population size and $\sigma^{\left(g\right)}$ is the step size.

Once the samples are drawn from this distribution, they are evaluated and ranked based on their objective function values. These ranked samples $x_{i:\lambda }^{\left(g+1\right)}$ are used to calculate $m^{\left(g+1\right)}$, $C^{\left(g+1\right)}$ and $\sigma^{\left(g+1\right)}$ of the next generation, using a set of update rules which are provided below.

Though CMA-ES is a highly effective optimization algorithm, its computational complexity is $O(d^{2})$ in space and time [23], where *d* is the dimension of the parameter vector to be optimized. Thus, the method is inefficient for solving high-dimensional problems where *d* is larger than a few hundreds.

### Appendix A.1. Hyperparameters

The CMA-ES uses several hyperparameters in order to perform optimization [63]. These include a damping parameter $d_{\sigma }$ and $c_{1},c_{\mu },c_{c},c_{m},c_{\sigma }$ which are “momentum”-like parameters, which control the amount of information retained from previous generations. Furthermore, $w_{i}$ are known as the “recombination weights”, which are used in most update rules. They are typically chosen such that

$$\sum_{j=1}^{\lambda }w_{j}\approx 0,\;\sum_{i=1}^{\mu }w_{i}=1,\;w_{1}\geq \ldots \geq w_{\mu }>0.$$

These are also used to calculate the effective population size for recombination: $\mu_{\mathrm{eff}}={\left(\sum_{i=1}^{\mu }w_{i}^{2}\right)}^{-1}$. For more details regarding the specific choices of these hyperparameters, please refer to [63].

### Appendix A.2. Mean Update Rule

As mentioned above, several update rules are employed in the CMA-ES. The first of those is the update of the mean $m^{\left(g+1\right)}$:

(A2) $$m^{\left(g+1\right)}=m^{\left(g\right)}+c_{m}\sum_{i=1}^{\mu }w_{i}\left(x_{i:\lambda }^{\left(g+1\right)}-m^{\left(g\right)}\right).$$

### Appendix A.3. Covariance Matrix Update Rule

The covariance matrix requires auxiliary vectors in order to construct its update rule; these are calculated using:

(A3) $$\begin{array}{ccc} p_{c}^{\left(g+1\right)} & = & \left(1-c_{c}\right)p_{c}^{\left(g\right)}+{\tilde{c}}_{c}\frac{m^{\left(g+1\right)}-m^{\left(g\right)}}{\sigma^{\left(g\right)}} \end{array}$$

(A4) $$\begin{array}{ccc} y_{i:\lambda }^{\left(g+1\right)} & = & \left(x_{i:\lambda }^{\left(g+1\right)}-m^{\left(g\right)}\right)/\sigma^{\left(g\right)}, \end{array}$$

where ${\tilde{c}}_{c}=\sqrt{c_{c}\left(2-c_{c}\right)\mu_{\mathrm{eff}}}$. These auxiliary vectors are then used to construct the rank-μ and rank-1 update matrices:

(A5) $$\begin{array}{ccc} C_{\mu }^{\left(g+1\right)} & = & \sum_{i=1}^{\lambda }w_{i}y_{i:\lambda }^{\left(g+1\right)}{\left(y_{i:\lambda }^{\left(g+1\right)}\right)}^{⊤} \end{array}$$

(A6) $$\begin{array}{ccc} C_{1}^{\left(g+1\right)} & = & p_{c}^{\left(g+1\right)}p_{c}^{{\left(g+1\right)}^{⊤}}. \end{array}$$

Finally, by defining $c_{old}=\left(1-c_{1}-c_{\mu }\sum_{i=1}^{\lambda }w_{i}\right)$, we obtain the update rule for the covariance matrix:

(A7) $$C^{\left(g+1\right)}=c_{old}C^{\left(g\right)}+c_{1}C_{1}^{\left(g\right)}+c_{\mu }C_{\mu }^{\left(g+1\right)}.$$

### Appendix A.4. Step-Size Update Rule

The last update rule is for the step size, which also requires the use of an auxiliary vector:

(A8) $$p_{\sigma }^{\left(g+1\right)}=\left(1-c_{\sigma }\right)p_{\sigma }^{\left(g\right)}+{\tilde{c}}_{\sigma }C^{{\left(g\right)}^{-\frac{1}{2}}}\frac{m^{\left(g+1\right)}-m^{\left(g\right)}}{\sigma^{\left(g\right)}},$$

where ${\tilde{c}}_{\sigma }=\sqrt{c_{\sigma }\left(2-c_{\sigma }\right)\mu_{\mathrm{eff}}}$. This is then used to construct the last update rule, which is for the step size:

(A9) $$\sigma^{\left(g+1\right)}=\sigma^{\left(g\right)}\exp\left(\frac{c_{\sigma }}{d_{\sigma }}\left(\frac{∥p_{\sigma }^{\left(g+1\right)}∥}{E∥N(0,I)∥}-1\right)\right),$$

where $E∥N(0,I)∥\approx \sqrt{n}+O\left(1/n\right)$ is the expectation of the $l_{2}$ norm of samples drawn from N(0,I).

### Appendix A.5. Next Generation

Once $m^{\left(g+1\right)}$, $C^{\left(g+1\right)}$ and $\sigma^{\left(g+1\right)}$ have been calculated, they are inserted into (A1) so that the next generation of samples can be drawn and the process repeated, until the convergence criteria are fulfilled.

## Appendix B. Computational Cost

The total number of epochs of our method is calculated as:

(A10) $$Total\_epochs=\mathrm{Pret}.\mathrm{epochs}+N_{Rounds}\cdot \left(N_{GF}+N_{GB}\right).$$

Additionally, the number of data samples used in each gradient-free step is:

(A11) $$\#data\_samples=M\cdot \frac{\#Batch}{S-Batch}\cdot Batch\_size,$$

where *M* is the number of CMA-ES samples in each gradient-free step, $\frac{\#Batch}{S-Batch}$ is the number of batches in each super-batch, and Batch_size is the number of data samples in each batch.

Using the experimental details for ImageNet from Table 1 in Equation (A10), we find that the GFB uses only 57 effective epochs, 30 quantization-aware training epochs and 27 epochs for the bit optimization (5 gradient-based epochs + 4 gradient-free steps, applied iteratively for 3 rounds: 27=3·(4+5)), and it requires the lowest computational cost compared to all the other methods, which typically require 100–200 epochs. Using Equation (A11) with the experimental details from Table 1, it can be shown that a gradient-based epoch uses 1.2 M data samples, while a gradient-free step uses 1.6 M data samples. However, gradient-free steps have a lower computational cost than gradient-based epochs because backpropagation is not performed during the gradient-free steps and because the internal CMA-ES operations have a negligible computational cost compared to the forward-pass operations of the networks. In practice, we find they have similar runtimes. For example, using our NVIDIA RTX2080-Ti, our training scheme applied to ResNet50 requires 4.91 GPU hours for a gradient-based epoch and 4.90 h for a gradient-free step.
